# Self-reported social and activity restrictions accompany local impairments in posterior tibial tendon dysfunction: a systematic review

**DOI:** 10.1186/s13047-018-0292-z

**Published:** 2018-08-30

**Authors:** Megan H. Ross, Michelle Smith, Melanie L. Plinsinga, Bill Vicenzino

**Affiliations:** Department of Physiotherapy, School of Health and Rehabilitation Sciences, University of Queensland, Brisbane, Australia

**Keywords:** Disability, Foot, Orthoses, Pain, Tendinopathy

## Abstract

**Background:**

Posterior tibial tendon dysfunction (PTTD) is a painful, progressive tendinopathy that reportedly predominates in middle-age, overweight women. There is no evidence based guidelines that clinicians can use to guide treatment planning, which leaves clinicians to make decisions on the basis of presenting clinical impairments and self-reported pain and disability. The purpose of this systematic review was to quantify clinical impairments, pain and disability in individuals with PTTD compared with controls.

**Methods:**

Five databases were searched for terms referring to the posterior tibial tendon and flatfoot up to and including 11 March 2018. The systematic review was registered with PROSPERO (CRD: 42016046951). Studies were eligible if they were published in the English language and contained data on clinical impairments, pain or disability compared between participants diagnosed with PTTD and pain-free individuals. Standardised mean differences (SMDs) were calculated where possible and meta-analysis was performed when homogeneity of outcomes allowed.

**Results:**

Ten eligible studies were identified and pooled in the meta-analyses. Strong effects were revealed for poor heel rise endurance (SMD -1.52, 95% CI -2.05 to − 0.99), less forefoot adduction-inversion strength (SMD -1.19, 95% CI -1.68 to − 0.71) and lower arch height (SMD -1.76, 95% CI -2.29 to − 1.23). Compared to controls, individuals with PTTD also had more self-reported stiffness (SMD 1.45, 95% CI 0.91 to 1.99), difficulties caused by foot problems (SMD 1.42, 95% CI 0.52 to 2.33) and social restrictions (SMD1.26, 95% CI 0.25 to 2.27).

**Conclusion:**

There is evidence of impaired tibialis posterior capacity and lowered arch height in individuals with PTTD compared to controls. Further to addressing the expected impairments in local tendon function and foot posture, pain, stiffness, functional limitations and social participation restrictions should be considered when managing PTTD.

**Electronic supplementary material:**

The online version of this article (10.1186/s13047-018-0292-z) contains supplementary material, which is available to authorized users.

## Background

Posterior tibial tendon dysfunction (PTTD) is a complex, progressive musculoskeletal disorder of the tibialis posterior tendon which most commonly affects mid-late aged women who frequently have systemic comorbidities [1–4]. Although data is limited, prevalence has been estimated to be 10% in older women [2], but is likely to be higher as PTTD often goes undiagnosed [2, 5]. The diagnosis of PTTD is most commonly made clinically, based on patient history (e.g. area of pain) and physical examination [6]. Key features of the physical examination are posterior tibial tendon pain on palpation or loading (e.g. weight bearing activities and heel raising) that is usually (but not always) accompanied with a flatfoot deformity, especially forefoot abduction (or the ‘too many toes sign’) [1, 7, 8]. Imaging is not routinely used in the diagnosis of PTTD, but when reported, it largely focuses on either the integrity of the tendon (ultrasound and MRI findings) [9, 10] or structural deformity of the foot (radiographic examination) [11, 12].

The non-operative management of this condition is usually advocated in the early stages and typically focuses on musculotendinous conditioning exercises and arch supporting devices (e.g. in-shoe foot orthoses and braces) [13–15]. There is a lack of high quality evidence for these treatments, which relegates physical therapy treatment decisions to one that targets presenting impairments and are based largely on the clinical reasoning skills of the clinician. This systematic review sought to comprehensively search the literature on physical impairments of PTTD. The primary research question for this systematic review was: Do individuals with PTTD have quantifiable differences in clinical impairments, pain and disability compared to controls? The secondary research question was: What is the relative magnitude of deficits in muscle function, foot posture and motion, pain and disability?

## Methods

The systematic review protocol was developed in accordance with the Preferred Reporting Items for Systematic Reviews and Meta-Analyses (PRISMA) statement [16] (Additional file 1) and registered online at http://www.crd.york.ac.uk/PROSPERO/display_record.asp?ID=CRD42016046951. Literature search criteria and methods were specified and agreed on in advance to minimise selection bias.

### Data sources and searches

An electronic database search was conducted across CINAHL, Cochrane, Embase, PubMed and Web of Science from database inception up to and including 11 March 2018, limited to the English language. The search strategy was broad to capture all relevant papers pertaining to past and present variations in terminology for the condition: flatfoot OR (posterior AND tibia* AND (tendon* OR tendin*)) OR “pes planus” OR “pes planovalgus”. The terms flatfoot, pes planus and pes planovalgus were included only to capture articles using varying terminology to describe PTTD; other causes of adult acquired flatfoot deformity (AAFD) and asymptomatic flatfoot were not included in this review. Due to limited literature available on the condition, a ‘participant’ (condition) only search was performed where articles were manually excluded based on intervention, comparator and outcome specifications.

### Study selection

Two independent reviewers (MHR and MLP) performed the search separately and results were imported into Endnote X7 (Thompson Reuters, Carlsbad, California, USA) where duplicates were removed. Titles and abstracts were screened for relevance by two reviewers (MHR and MLP), with disagreements resolved by consensus with reference to a third reviewer (BV). Full text versions of remaining articles were obtained and screened against final eligibility criteria by two reviewers (MHR and MLP).

Studies were eligible for inclusion if they were published in the English language and contained data on clinical impairments, pain or disability compared between participants diagnosed with PTTD (or AAFD related to tendon dysfunction) and pain-free individuals. Studies including participants who had undergone a specific intervention were included only if baseline or pre-intervention data was reported and compared to control participants without the condition. Any post-intervention data was not included.

Studies were excluded if there was no comparison group or clinical measures of pain, function or disability, the study was published in a language other than English, or the full text was not available. Review articles, single case reports, paediatric, cadaver and animal studies were excluded. Studies including participants with other conditions such as osteoarthritis or rheumatoid arthritis that did not include separate data for individuals with PTTD or AAFD were also excluded.

### Data extraction and quality assessment

Where available, the following information was extracted from all eligible studies: study design, recruitment source, inclusion/exclusion criteria, sample size, stage of PTTD [1], population characteristics and comparison group characteristics. Quantitative data relating to outcome measures for physical impairment, pain and disability, specifically mean SD for continuous outcomes, were extracted to enable calculation of effect size. Data extraction was performed by two independent reviewers (MHR and MLP) and recorded in a pre-determined spreadsheet. Corresponding authors were contacted for additional information when reported data was insufficient for analyses. A third reviewer (MS) verified data extraction prior to analysis.

Methodological quality of included articles was evaluated using the Epidemiological Appraisal Instrument (EAI), which has been shown to be a valid and reliable tool for the assessment of observational studies [17]. Twenty-one items from the original EAI were used following removal of items that were not applicable to cross-sectional and case-control study designs. Removed items specifically related to interventions, randomisation, follow-up period and environmental factors. Detailed criteria for each response were clarified a-priori to match the purpose of this review.

Two independent assessors (MHR and MLP) rated all included articles. Where a consensus was not able to be reached, disagreements were resolved by a third investigator (BV). Each item was scored as either “Yes” (score = 1), “Partial” (score = 0.5), “No” (score = 0), “Unable to determine” (score = 0) or “Not Applicable” (item removed from scoring) and an overall score was derived as an average score across all applicable items (range = 0 to 1).

### Data synthesis

Reliability of the methodological quality assessment was calculated in Stata v13 (College Station, TX: StataCorp LP). The *ĸ* statistic (95% CI) was used to report the inter-rater reliability of the quality ratings between the two assessors. The *ĸ* statistic was interpreted as < 0.00 poor agreement; 0.00–0.20 slight agreement, 0.21–0.40 fair agreement, 0.41–0.60 moderate agreement, 0.61–0.80 substantial agreement, and 0.81 to 1.00 almost perfect agreement [18].

### Analysis

Standardised mean differences and 95% CIs were calculated for continuous variables in Review Manager (RevMan) V5.3 (Copenhagen: The Nordic Cochrane Centre, The Cochrane Collaboration) using random effects models. SMDs were calculated as the difference between PTTD and control group means, divided by the pooled SD [19]. Where 95% CIs did not contain zero, the difference between groups was considered statistically significant. For each outcome measure, a positive SMD reflected greater values in the PTTD population and a negative SMD reflected greater values in the control population. Effect sizes were interpreted based on Hopkins, as follows; < 0.2 trivial effect, 0.2–0.6 small effect, 0.61–1.2 medium effect, and > 1.2 large effect [20].

Meta-analysis was performed where similar methodology and outcome measures (study homogeneity) allowed pooling of data. Chi-squared tests (*p* < 0.1) and the *I*^*2*^ statistic were used to quantify study heterogeneity for pooled SMDs [21] with ≥0.75 considered substantial heterogeneity. A summary of main findings and study conclusions were presented where data were not available to calculate SMDs.

## Results

### Flow of studies through the review

**Fig. 1:**
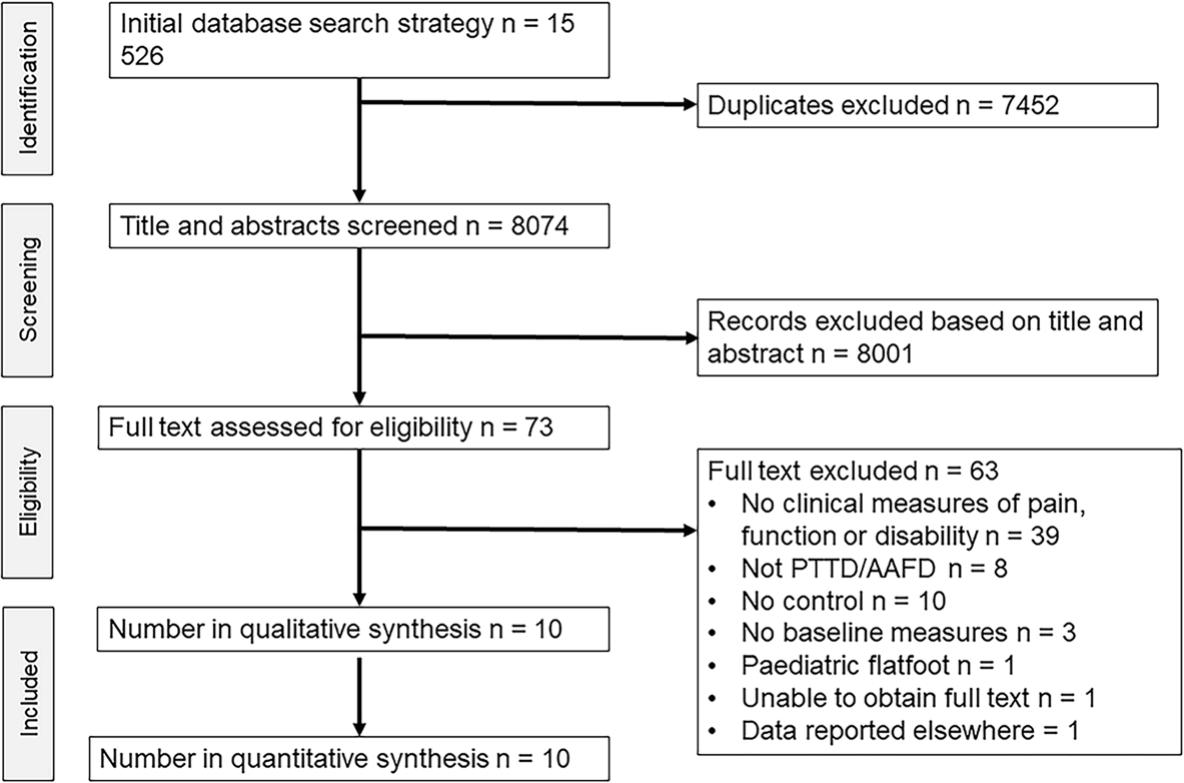
Flow of studies through the review

The search strategy identified a total of 15,526 articles of which 7452 were removed as duplicates (Fig. 1). The remaining 8074 articles were screened by title and abstract and 73 potentially eligible articles were identified. Full text screening of the 73 articles excluded 63 articles that did not meet the inclusion criteria (Fig. 1). The 10 remaining articles underwent methodological quality assessment and data extraction. Four authors were contacted for additional data for five articles. Data from two studies was made available [22, 23] but not from others [24–26] with reasons being that the data was not collected or not available. Papers that reported on the same population sample were only included once in the analysis. One author was contacted to clarify that two papers [22, 27] reported data from the same sample, and as no additional (unique) data was provided, the second paper was excluded [27].

### Quality assessment

Overall agreement on methodological quality of included studies was almost perfect (absolute agreement = 98.64%, *ĸ* = 0.97, 95% CI 0.85 to 1.00). Agreement was reached on 215 out of 220 EAI items in total. Consensus was obtained on the quality rating of the five remaining items. Overall EAI scores ranged from 0.500 to 0.682 out of a possible score of 1 (Table 1). The methodological quality assessment revealed that only two studies (20%) adequately reported the source of the participant population, 20% performed sample size calculations and 40% had a control group adequately comparable to the case group for important characteristics that could otherwise confound the findings (e.g. age, sex, etc.). The reliability and validity of outcome measures were reported by 30% and 10% of studies respectively. One study collected data on duration of symptoms yet no studies (0%) accounted for history of symptoms in analyses. Generalisability of results to other populations was low (0%); 6 studies reported samples of convenience and the remaining 4 studies reported data for participants seeking treatment for their condition (referral from clinics).

**Table 1:** Results from quality assessment of all included papers (*n* = 10) on the EAI

	Reference	Kulig ^26^	Rabbito ^28^	Neville ^22^	Houck ^24^	Chimenti ^25^	Neville ^23^	Kulig ^32^	Houck ^30^	Tome ^31^	Houck ^29^	Studies scoring “yes” (%)
Q1.	Hypothesis/aim/ objective clearly described	1	1	1	1	1	1	1	1	1	1	100
Q2.	Main outcomes clearly described	1	1	1	1	1	1	1	1	1	1	100
Q3.	Reported study design	1	1	0.5	0.5	1	1	1	1	1	1	80
Q4.	Source of participant population clearly described	1	1	0	0	0	0	0.5	0	0	0	20
Q5.	Reported eligibility criteria	1	1	1	1	1	1	1	1	1	1	100
Q6.	Characteristics of study participants described	1	1	1	1	1	1	1	1	1	1	100
Q7.	Important covariates and confounders described	1	1	1	1	1	1	1	1	1	1	100
Q8.	Statistical methods clearly described	1	1	1	1	1	1	1	1	1	1	100
Q9.	Main findings of the study clearly described	1	1	1	1	1	1	1	1	1	1	100
Q10.	Provides estimates of the random variability in the data	1	1	1	1	1	1	1	1	1	1	100
Q11.	Sample size calculations	1	1	0	0.5	0.5	0	0	0	0	0	20
Q12.	Comparability of case/control groups	0	1	1	1	1	0	0	0	0	0	40
Q13.	Recruitment period for case/control groups	0	0	0	0	0	0	0	0	0	0	0
Q14.	Blinding of assessors	0	0	0	0	0	0	0	0	0	0	0
Q15.	Reliability of outcome measures	1	0.5	1	1	0	0.5	0	0.5	0.5	0	30
Q16.	Validity of outcome measures	1	0	0	0	0	0	0	0	0	0	10
Q17.	Standardised assessment	1	1	1	1	1	1	1	1	1	1	100
Q18.	Assessment period of case/control groups	0	0	0	0	0	0	0	0	0	0	0
Q19.	History of disease/symptoms collected and included in analysis	0	0	0.5	0	0	0	0	0	0	0	0
Q20.	Adjusted for covariates	1	1	1	1	0.5	1	1	1	1	1	90
Q21.	Reported data for subgroups	0	0	1	0	0	1	0	0	0	0	20
Q22.	Generalibility of results to other populations	0.5	0.5	0.5	0.5	0.5	0	0.5	0	0	0	0
Overall quality score (range 0 to 1)		0.70	0.68	0.66	0.61	0.57	0.57	0.55	0.52	0.52	0.50	0.59

Key: 1 =” Yes”, 0.5 = “Partial”, 0 = “No” or “Unable to determine”

*Abbreviations: EAI* epidemiological appraisal instrument

### Participants

The 10 included studies contained a total of 213 participants with PTTD compared to 144 healthy controls. Sample sizes ranged from 12 [28] to 30 [22, 29, 30] PTTD participants (Table 2) and 10 [23, 31] to 20 [32] controls. Mean (SD) age of PTTD patients ranged from 30.3 (7.9) [28] to 61.0 (10.0) [24] years and the proportion of females ranged from 63.3% [22] to 100.0% [26, 32].

**Table 2:** Study design, PTTD diagnosis, clinical impairments and participant characteristics, which are presented as mean (SD) or count (percentage)

Study ID	Study design	Diagnosis	Selection criteria for PTTD	Clinical impairments	PTTD				Control			
					n	Female (%)	Age years	BMI kg/m	n	Female (%)	Age years	BMI kg/m
Chimenti ^25^	Cross-sectional laboratory	Stage II AAFD	1 or more signs of tendinopathy (tenderness, swelling or pain with unilateral heel raise) and 1 or more signs of flexible flatfoot deformity (excessive non-fixed hindfoot eversion, excessive first metatarsal abduction or loss of medial longitudinal arch height)	Function & strength, Foot posture, PROM	20	14 (70)	57 (11.3)	30 (5.2)	15	11 (73)	56 (5.3)	26 (4.4)
Houck ^30^	Cross-sectional laboratory	Unilateral stage II PTTD	1 or more signs of tendinopathy (tenderness, swelling or pain with unilateral heel raise) and 1 or more signs of flexible flatfoot deformity (excessive non-fixed hindfoot eversion, excessive first metatarsal abduction or loss of medial longitudinal arch height)	Foot posture	30	22 (73)	59.3 (10.8)	29.6 (4.8)	15	14 (93)	56.5 (7.7)	30.5 (3.6)
Houck ^29^	Cross-sectional laboratory	Unilateral stage II PTTD	1 or more signs of tendinopathy (tenderness, swelling or pain with unilateral heel raise) and 1 or more signs of flexible flatfoot deformity (excessive non-fixed hindfoot eversion, excessive first metatarsal abduction or loss of medial longitudinal arch height)	Function & strength, Foot posture	30	21 (70)	59.8 (11.1)	29.9 (4.8)	15	14 (93)	56.5 (7.7)	30.6 (3.6)
Houck ^24^	Case-control	Unilateral stage II PTTD	Signs of tendon pathology (pain and/or swelling along medial ankle) and flexible flatfoot deformity (hindfoot eversion, forefoot abduction or loss of medial longitudinal arch height)	Function & strength, Foot posture	24	18 (75)	61 (10)	30 (5)	15	13 (87)	55 (8)	28 (5)
Kulig ^26^	Cross-sectional laboratory	Unilateral early stage PTTD (I or II)	Pain along medial ankle, tender on palpation posterior tibial tendon, lowered medial longitudinal arch, abducted midfoot, absence of rigid foot deformity	Function & strength, Foot posture, PROM	17	17 (100)	52.1 (7.5)	29.5 (6.3)	17	17 (100)	50.7 (5.5)	26.9 (5.9)
Kulig ^32^	Case-control	Unilateral early stage PTTD (I or II)	Pain along medial ankle, tender on palpation posterior tibial tendon, lowered medial longitudinal arch, abducted midfoot, absence of rigid foot deformity	Function & strength, Foot posture, Balance	19	19 (100)	54.6 (6.3)	28.9 (4.5)	20	20 (100)	50.8 (5.5)	26.9 (5.9)
Neville ^22^	Cross-sectional	Unilateral stage II PTTD	1 or more signs of tendinopathy (tenderness, swelling or pain with unilateral heel raise) and 1 or more signs of flexible flatfoot deformity (excessive non-fixed hindfoot eversion, excessive first metatarsal abduction or loss of medial longitudinal arch height)	Function & strength, ROM, Foot posture	30	19 (63)	58.1 (10.5)	30.6 (5.4)	15	14 (93)	56.5 (7.7)	30.6 (3.6)
Neville ^23^	Case-control	Unilateral stage II PTTD	1 or more signs of tendinopathy (tenderness, swelling or pain with unilateral heel raise) and 1 or more signs of flexible flatfoot deformity (excessive non-fixed hindfoot eversion, excessive first metatarsal abduction or loss of medial longitudinal arch height)	Foot posture, PROM	17	14 (82)	56.1 (11.6)	33.2 (7.4)	10	7 (70)	50.2 (6.8)	31.8 (3.8)
Rabbito ^28^	Case-control	Stage I PTTD	Mild swelling, tenderness, pain posterior to the medial malleolus, aggravated by recreational activity	ROM, Foot posture	12	9 (75)	30.3 (7.9)	23.2 (3.4)	12	9 (75)	28.5 (8.6)	23.7 (2.8)
Tome ^31^	Case-control	Unilateral stage II PTTD	1 or more signs of tendinopathy (tenderness, swelling or pain with unilateral heel raise) and 1 or more signs of flexible flatfoot deformity (excessive non-fixed hindfoot eversion, excessive first metatarsal abduction or loss of medial longitudinal arch height)	Foot posture	14	12 (85)	56.8 (11.7)	33.7 (7.4)	10	7 (70)	51.2 (7.3)	31.8 (3.6)

*Abbreviations: AAFD* adult acquired flatfoot deformity, *PTTD* posterior tibial tendon dysfunction, *ROM* range of motion, *PROM* patient reported outcome measure

Table 2 has details of the stage and criteria by which participants with PTTD were selected. In brief, one study investigated stage I PTTD [28], two studies investigated stage I-II PTTD [26, 32] and the remaining seven studies investigated stage II PTTD only [22–25, 29–31]. The method of diagnosis was by clinical assessment in all studies with 9 out of 10 studies requiring both signs of tendon pathology and flexible flatfoot deformity for a positive diagnosis. The one study investigating stage I PTTD [28] required only signs of tendon pathology including mild swelling and/or tenderness posterior to the medial malleolus that had been present for at least 3 weeks and aggravated by recreational activity.

### Outcome measures

Outcome measures reported for clinical impairments included heel raise performance [25, 26, 29, 32], leg muscle strength [22, 24], ankle range of motion [22, 28], hip muscle function [26], foot posture [22–26, 28–32], single leg balance [32] and distance walked and pain experienced during the 6-min walk test (6MWT) [26]. Pain was reported as an outcome measure following the 6MWT [26]. Patient-reported outcome measures included the Foot Function Index-Revised (FFI-R) [25, 26] and the Short Musculoskeletal Functional Assessment [23]. Meta-analysis was able to be conducted for a total of eight outcome measures.

### Main findings

#### Heel raise performance

**Fig. 2:**
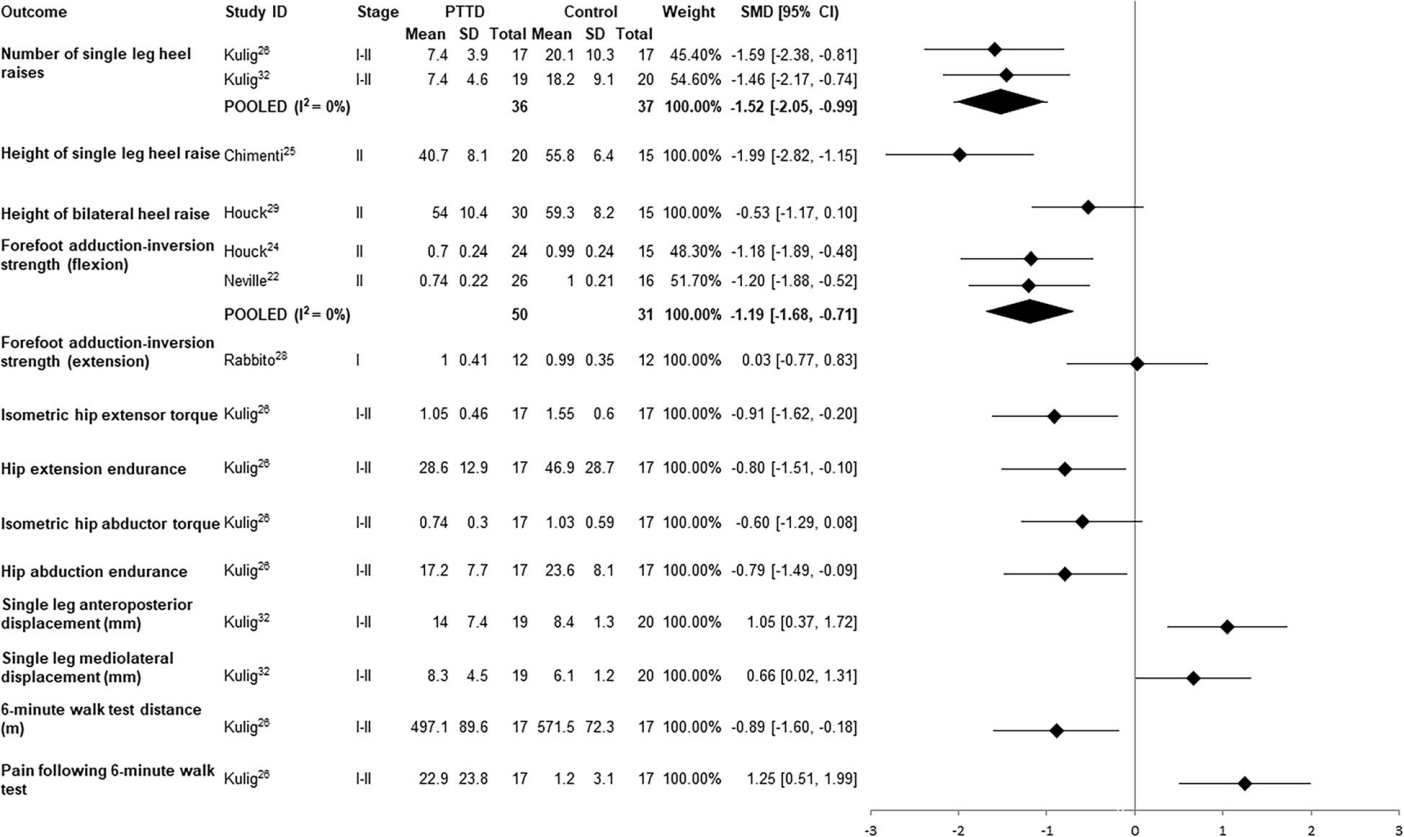
Standardised mean difference (95% CI) for function and strength outcomes in PTTD vs controls

Two clinical measures of heel raise performance (maximum number completed and height) were reported across four studies. Two studies were pooled and found a large effect size for the number of single leg heel raises performed by individuals with PTTD compared to controls (i.e. approximately 7 v 20 respectively; Fig. 2) [26, 32]. One study reported significantly lower height on single leg heel raise [25], whereas another reported no differences for bilateral heel raise height between PTTD and control groups (Fig. 2) [29].

#### Leg muscle strength

Combined isometric forefoot adduction and subtalar inversion strength in plantar flexion was reported in three studies [22, 24, 28]. Pooled data from two studies that measured strength in 90 degrees of knee flexion [22, 24] revealed a moderate deficit (based on an SMD value of − 1.19) in PTTD compared to controls (MD − 0.27 N/kg) (Fig. 2). The other study measured forefoot abduction and subtalar inversion strength in full knee extension [28] and showed no difference (MD 0.01 N/kg). It was excluded from the pooled analysis due to heterogeneity of testing position.

#### Hip muscle function

Hip extensor and abductor muscle strength and endurance in individuals with PTTD were compared to controls in one study [26]. Large SMDs indicate that participants with PTTD had significantly reduced hip extensor strength and endurance compared to controls (Fig. 2). There was a small-moderate effect for hip abductor muscle strength differences between PTTD and control groups, which did not reach statistical significance. SMDs for hip abductor muscle endurance revealed a significant medium effect with control participants demonstrating greater hip abductor muscle endurance than PTTD participants.

#### Single leg balance

Anteroposterior and mediolateral centre of pressure displacement during single leg stance was moderately greater in participants with PTTD compared to control (Fig. 2) [32]. The same study reported that 47% (9/19) of participants with PTTD were unable to maintain single leg balance for 10 s compared with 15% of controls (3/20) [32].

#### 6-min walk test

One study measured distance walked in 6 min (6MWT) and pain experienced on a 100 mm visual analogue scale [26]. Participants with PTTD covered a significantly shorter distance (approximately 74 m) and reported a significantly higher pain level (22 mm on visual analogue scale) when compared to individuals without PTTD (Fig. 2).

#### Foot posture

**Fig. 3:**
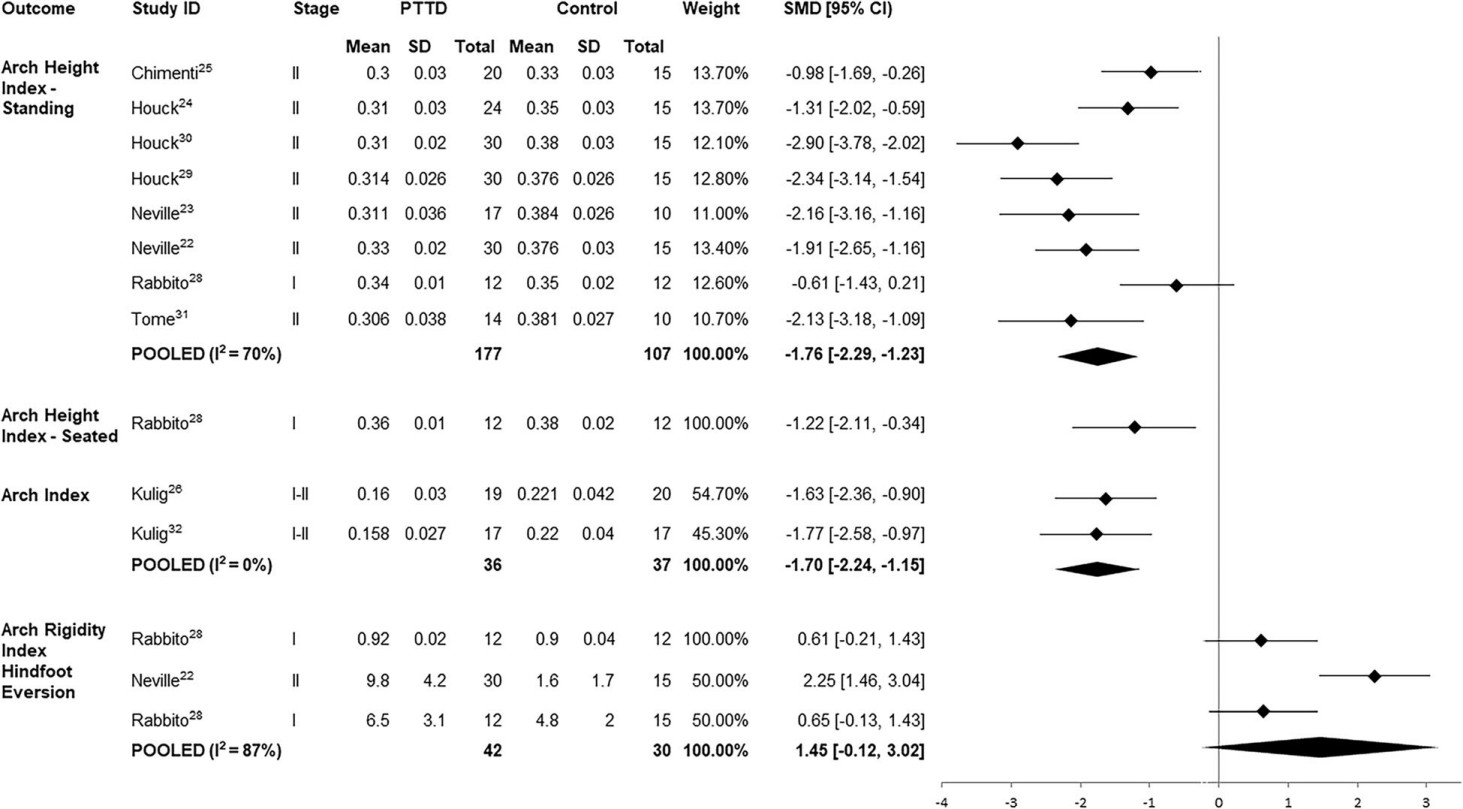
Standardised mean difference (95% CI) for foot posture and range of motion outcomes in PTTD vs controls

Foot posture was examined in two studies by using the Arch Index (AI) [26, 32] and in eight studies using the Arch Height Index (AHI) [22–25, 28–31]. Pooled SMDs for the two studies investigating AI, [26, 32] revealed a significant large effect indicating that PTTD participants demonstrated a flatter foot posture compared to controls. AHI in bilateral stance was substantially (large SMD) lower in individuals with PTTD compared to controls (Fig. 3) [22–25, 28–31]. There was a large SMD for AHI taken in a seated position, yet the Arch Rigidity Index (ratio of standing AHI to seated AHI) was not different between PTTD and control groups (Fig. 3) [28].

#### Hindfoot range of motion

Two studies measured hindfoot eversion range of motion [22, 28] and while the pooled SMD was large, reflecting more eversion in PTTD compared to controls, this was not statistically significant (confidence intervals contained 0) (Fig. 3).

#### Self-reported function

**Fig. 4:**
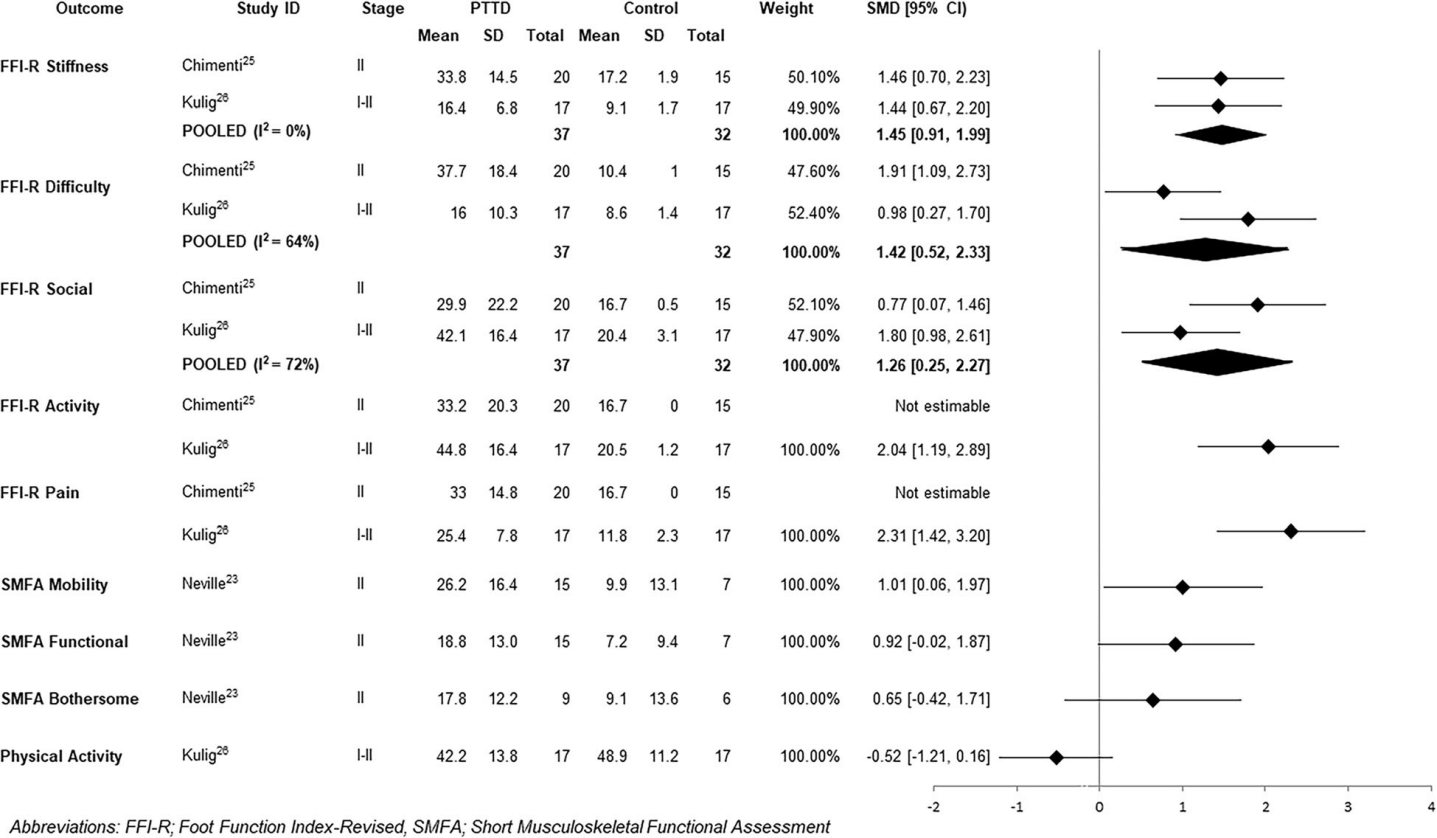
Standardised mean difference (95% CI) for patient-reported outcome measures

Five studies investigated self-reported function compared to controls using the Foot Function Index-Revised (FFI-R) [25, 26] and the Short Musculoskeletal Functional Assessment [23]. Pooled SMDs were calculated for the stiffness, difficulty and social subscales of the FFI-R with large effect sizes demonstrating significantly more self-reported stiffness, difficulty and social restrictions in individuals with PTTD (Fig. 4). As one study reported an SD of 0 for the pain and function subscales, pooled SMDs were not able to be calculated [25]. Another study [26] revealed that compared to controls, participants with PTTD had significantly higher self-reported pain and activity limitations (Fig. 4).

Participants with PTTD demonstrated significantly more self-reported mobility difficulties (Fig. 4) than controls on the Short Musculoskeletal Functional Assessment [22]. No significant differences between groups were found for functional limitations or the bothersome index (Fig. 4).

Levels of self-reported physical activity were not significantly different between individuals with PTTD and controls (Fig. 4) [26].

## Discussion

This is the first review to systematically evaluate and synthesise results of research investigating clinical impairments and self-reported pain and disability associated with PTTD. Data from meta-analyses indicate strong evidence for lower arch height and a lesser capacity to perform repeated unilateral heel rise in individuals with PTTD. These deficits align with the function of posterior tibialis muscle, which is governed by its orientation and attachments. A large effect size for a deficit in single leg heel rise height and a medium effect for combined isometric forefoot adductor and subtalar invertor muscle strength in plantar flexion from individual studies further supports impaired muscle function in PTTD.

While meta-analysis revealed strong evidence for lower arch height in individuals with PTTD compared to controls. The magnitude of this effect must be interpreted with caution because control participants in five studies were only included if they had normal AHI and visually assessed normal foot posture [22, 23, 29–31]. A requirement for pain-free individuals to demonstrate normal AHI and foot posture may have potentially magnified the effect seen between PTTD and controls. A finding that mitigates against this over-estimate of effect is that there was a large effect size of lower foot arch height in two studies that did not require controls to demonstrate normal foot posture. This suggests that key features of PTTD is likely a combination of both impaired muscle function (as discussed above) and postural deformity.

Impairments demonstrated in PTTD compared to controls were not limited to the level of body structure and function; lower self-reported function and greater pain also appear to be characteristic of PTTD. Meta-analyses of FFI-R data suggest that stiffness, functional difficulties and social limitations are key features of PTTD, with individual study SMDs also showing large effects for pain and activity limitations. Activity limitations were also not limited to self-report measures; poorer balance and mobility were demonstrated in PTTD compared to controls with a moderate effect. The deficit in physical capacity (heel raise number and height. and plantar flexion inversion weakness) and concomitant self-report concerns in functional, social, and activity limitations as well as pain ought to be considered in the management of the condition.

Clinical impairments in PTTD are not limited locally to the foot and ankle. Medium effects were found for deficits in hip extension strength and endurance and hip abduction endurance in individuals with PTTD [26]. Hip abduction strength deficits did not reach statistical significance (SMD -0.60, 95% CI-1.29, 0.08) yet sample size was small and this may reflect a type II error. While further research is needed to determine true effects, these results are consistent with findings of impaired hip muscle function in other distal joint pathologies of the lower limb including knee osteoarthritis [33], patellofemoral pain [34–36] and midportion achilles tendinopathy [37]. These data suggest the need to assess and consider addressing any potential deficits in hip muscle capacity in the management of patients with PTTD.

All studies included within this review pertained to either stage I (n of studies =1), II (*n* = 6) or I-II (*n* = 2) PTTD with data combined for analysis. Data for stage I and II PTTD were pooled for two meta-analyses; hindfoot eversion and AHI. Considering hindfoot eversion, one study that investigated stage II PTTD found strong evidence for increased hindfoot eversion ROM [22], whereas differences between individuals with stage I PTTD and controls were less prominent (Fig. 2) [28]. Similarly, seven of the eight papers investigating AHI found significant medium to large effects for lower AHI in stage II PTTD compared to controls, whereas AHI in stage I PTTD [28] did not appear to be different when compared to controls. When data for these outcomes were pooled, there was substantial heterogeneity (*I*^*2*^ = 87% and 70% respectively) and wide 95% confidence intervals, which makes it difficult to draw conclusions about the true effects. The variability observed may be a result of underlying differences between stage I and II PTTD and as such, the results must be interpreted with caution.

Variations in participant characteristics, including age, BMI and physical activity participation, between studies investigating stage I and II PTTD need to be considered in terms of contribution to some of the differences observed in the outcomes reported in this systematic review. Participants in the study that investigated stage I PTTD were younger [28] and had a markedly lower BMI [28] than those in the studies that investigated stage II PTTD (Table 2). Age and BMI for participants in two studies investigating stage I-II PTTD [26, 32] sat between those reported for stage I and stage II separately. All participants in the study that investigated stage I PTTD were undertaking running and running-related activities for at least 30 min three times per week [28]. While physical activity participation was not reported in most stage II studies, individuals with stage II PTTD were found to have significant activity limitations compared to controls based on the FFI-R activity subscale.

As PTTD is considered a progressive condition [1], younger, active individuals with stage I PTTD may not yet have progressed to a point where they present with certain signs of the condition, such as flatfoot deformity or an everted hindfoot, that may be more apparent in stage II PTTD. In line with classification systems [1, 7, 8, 38] and consistent with other studies [39], this suggests that changes in foot posture may not be a key feature of stage I PTTD. Differences between stage I and II PTTD also appear to relate to muscle function. In stage I PTTD, no difference was found for ankle inverter strength compared to controls [28]. This is in direct contrast to results from stage II studies that found strong evidence for lower isometric forefoot adduction and subtalar inversion strength in individuals with stage II PTTD compared to controls. This suggests that while pain is a feature in both stages of PTTD, the tibialis posterior muscle is likely more competent in stage I of the condition.

There are a number of factors to consider when interpreting results of this systematic review. While no restrictions were made regarding the stage of condition, these results apply to only stage I and II PTTD as no data was available for stage III or IV. Without quantifiable methods for staging the condition [40], delineation between stages must be interpreted with caution. While all studies reported eligibility criteria relating to stage I or II PTTD (100% on the quality appraisal), assessment of stage was based on classification systems that have not been validated [40]. Clinical differentiation between stage II and III PTTD has been based on the widely accepted notion that stage II is a flexible deformity, whereas in stage III the deformity is fixed [1]. The problem with this is that the method used to determine flexibility of the deformity is not reported. Perhaps this is an omission in reporting but it is more likely due to the lack of a valid clinical method of quantifying flexibility. Future research investigating clinical tools that may be able to provide a valid and reliable method of determining the stage of the condition would be beneficial for clinicians and academics.

Another consideration is that this review was limited to 10 studies with relatively small sample sizes. The outcome with the strongest effect was based on a sample of 177 individuals with PTTD and 107 controls. The majority of outcomes had a sample size much smaller than this, and were calculated from individual papers. Small sample sizes and heterogeneity among included studies suggests effect estimates should be interpreted with caution. While SMDs were calculated in this review where possible to overcome small sample sizes, the current small body of PTTD literature would benefit from larger, well-designed studies.

## Conclusion

This review has appraised the existing literature and shows that PTTD is characterised by impairments related to both local tendon dysfunction and foot posture as expected. However, the condition is also associated with changes in hip strength, walking, balance and global measures of self-reported function. These results highlight the need to consider both local impairments and measures of overall function when assessing the presentation and impact of the condition clinically, the effectiveness of PTTD management, and when designing future studies.

## Additional file

Additional file 1: PRISMA Checklist. (DOC 62 kb)

## Abbreviations

6MWT: 6-min walk test
AAFD: Adult acquired flatfoot deformity
AHI: Arch height index
AI: Arch index
CI: Confidence interval
EAI: Epidemiological appraisal instrument
FFI-R: Foot function index-Revised
PTTD: Posterior tibial tendon dysfunction
SMD: Standardised mean difference
